# Challenges in nature-based health and therapy research and critical considerations for application in musculoskeletal health

**DOI:** 10.3389/fpubh.2025.1509419

**Published:** 2025-01-22

**Authors:** Richard Doran-Sherlock, Payal Sood, Nicole Anne Struthers, Filip Maric

**Affiliations:** ^1^Registered Osteopath, Dublin, Ireland; ^2^School of Health and Social Care, Swansea University, Swansea, Wales, United Kingdom; ^3^Health and Rehabilitation Sciences, Faculty of Health Sciences, Western University, London, ON, Canada; ^4^Faculty of Health Sciences, Department of Health and Care Sciences, UiT The Arctic University of Norway, Tromsø, Norway

**Keywords:** nature-based therapy, nature-based interventions, planetary health, physical health, musculoskeletal health

## Abstract

Nature-based health and therapy (NBHT) is a term incorporating a broad suite of practices that focus on engagement with the natural world and nature-rich spaces for potential physical and mental health benefits. As healthcare professions such as physiotherapy and osteopathy move away from biomedical/reductionist models of care for complex conditions towards approaches which take into account social and environmental determinants of health, NBHT may become part of clinical interventions and public health messaging. However, there are multiple challenges in aspects of NBHT research and application, from methodological issues in the primary research base, to questions of environmental injustice and access inequalities in many areas. In addition, engaging with natural environments which are vulnerable to the entwinned threats of climate change and biodiversity collapse requires consideration of the effects of ecological disturbance and the underlying anthropocentric/utilitarian view of the natural world. In this perspective, we outline a critique of NBHT literature and offer positive suggestions for how better-quality research can be conducted and implemented by focusing on local environmental, social, and political factors. We conclude by outlining a set of critical considerations that healthcare professionals might use to develop and implement NBHT programmes in their specific regional contexts.

## Introduction

Nature-based health and therapy (NBHT) refers to a wide range of practices involving engagement with nature-rich spaces or stimuli to elicit potential mental and physical health benefits (1). The individual practices which comprise NBHT can vary from *Shinrin yoku* (forest bathing), a form of structured relaxation in a forested environment first developed in Japan in 1982 (2), to gardening/horticulture (3), or wilderness exploration (4). Without adapting a specific interventional label, spending time in urban greenspace (parks, woodlands, etc.) is increasingly considered an important aspect of public health, and ensuring equality of access is considered to be a social justice issue (5). Research into this subject has been growing, with multiple systematic reviews now published focusing on the potential impact of NBHT on issues ranging from physical health conditions (6), to depression (7) and stress (8). Although the field is relatively novel, there have been calls to develop the research base and implementation of NBHT based programmes in public health and planning policy (9), while health bodies such as the United Kingdom’s National Health Service have committed to further implementing NBHT in social prescription (10).

Three theories have been frequently cited as underpinning the potential health benefits of NBHT: Attention Restoration Theory, a cognitive model in which natural spaces reduce the demand on voluntary attentional control mechanisms (11), Stress-Reduction Theory (SRT) in which experiences with nature are believed to trigger emotional and physiological changes (12), and the Biophilia Hypothesis (13) which posits that humans have an innate attraction towards living entities and processes (14). While these theoretical frameworks are subject to debate based on their evolutionary assumptions and purported mechanisms (15–17), epidemiological data suggests that exposure to natural environments is associated with positive health outcomes. Systematic reviews have linked greenspace with increased levels of physical activity, improved mood, and decreased overall mortality (5, 18), but most studies which comprise this research involve cross-sectional designs limiting the ability to infer causality. Research on blue spaces (rivers, lakes, shores, etc.) reveals similar trends, with positive associations between proximity to blue space and levels of physical activity (19), mental health metrics (20), and a negative association with all-cause mortality (21). Although individual effect sizes are generally small (21), and spatial factors such as accessibility, usability, visibility and ecological quality are not sufficiently accounted for (5, 22, 23), the literature is broadly consistent in showing statistically significant positive health outcomes with green/blue space exposure. Structuring this exposure through NBHT is a potential avenue for health interventions by multiple specialities.

Healthcare professions such as physiotherapy and osteopathy, for whom musculoskeletal (MSK) issues are a central focus, have traditionally based their practice on biomedical models of health and disease (24, 25). The traditional biomedical perspectives viewed biological or anatomical factors as determinants of the development of pathology or disease (26, 27). In recent decades, research has challenged these models, as complex conditions that fall under the professional scope of practice, such as persistent MSK pain, are poorly explained or treated by reductionist approaches (28–30). To address the multifactorial and interrelated nature of pain, the biopsychosocial (BPS) model has been used as a framework to understand complex conditions as emergent states arising from the interaction of biological, emotional/psychological, and social domains (31, 32). While the BPS model has led to promising developments in research and practice (33), it has also been argued that the general understanding and implementation of the model remains reductionist and particularly social and environmental factors are not yet considered sufficiently (34–36).

The role of environmental factors, defined as the external factors constituting the physical, social and attitudinal environment influencing the experience and management of a health condition, has been emphasised by the World Health Organisation (37) in The International Classification of Functioning, Disability, and Health Framework. Somewhat in line with this, Lehman et al. (38) have argued for the need to emphasise the role of contextual dynamics play in the BPS model because environmental factors have significant positive (e.g., natural spaces facilitating movement/exercise) and negative (e.g., air pollution) influences on health. Because of the limited theoretical foundations and problematic applications of the BPS model, others have argued for an enactive-ecological model that more clearly advances an understanding of the body as inseparable from its social and ecological environment (34, 36, 39). Insofar the external environment is an important factor in either of these frameworks, environmental interventions such as NBHT may modify BPS model stressors or offer affordances for salutogenic behaviours. As such, healthcare practitioners and organisations engaging with people living with complex conditions may want to consider their potential role in prescribing, designing, facilitating, or lobbying for NBHT programmes. In doing so, it is important that individuals and organisations are cognisant of some inherent challenges to research and application of this field. The purpose of this perspective paper is to outline some of the key challenges while offering a set of critical considerations to aid in the design of NBHT interventions across a range of ecological and social contexts.

## Challenges in NBHT research

While the field of NBHT is growing, many of the primary studies are of poor methodological quality, and as such, it is important to be cautious about any stated benefit arising from nature interaction [e.g., (6, 7)]. While most systematic reviews found a small but statistically significant impact of NBHT on mood and stress, improved study protocols are necessary to reduce uncertainty in this field (8, 40). Several factors contribute to this uncertainty – studies with high risk of bias, small sample sizes, heterogeneity of study design, etc. – and a major limitation for generalising the results is the frequent use of self-referred subjects who may be recruited due to a pro-nature disposition (8). Additionally, many studies measuring psychological outcomes are focused on asymptomatic populations (40), making extrapolation to people with specific diseases and disorders difficult.

In addition to the latter, the challenges of studying complex conditions and complex interventions must also be considered. Many of the conditions that NBHT seeks to impact (depression, anxiety, pain, stress, etc.) may be best understood within the context of the BPS model (38, 41). As such, research that attempts to establish a causal relationship between the dose of NBHT and response (e.g., symptom modification) may be overly reductive in its assumptions about the condition.

It could also be argued that NBHT is a complex intervention (42), as it entails multi-sensory stimuli, multiple potential mechanisms of action (43), and psychosocial moderators and mediators of effect (44). The challenges of studying complex interventions for multifactorial conditions are not trivial, and NBHT may have similar issues to address as more widely used MSK interventions like manual therapy and exercise (45, 46). Hansen et al. (47) and Doran-Sherlock et al. (7) identified a lack of qualitative and mixed-method studies in this field, and future corresponding research may help to illuminate how NBHT can be applied for distinct individuals and groups. Although improved methodological standards and a wider research framework are essential, following the BPS model and enactive-ecological models, it is also important to consider that the intrinsic, non-linear coupling between the body, brain, and external world (41, 48) can be a barrier to quantifying individual outcomes from NBHT.

## Challenges in the implementation of NBHT

While addressing issues with the methodological and theoretical quality of the research base, it is important to consider how and why NBHT can produce varying effects on individuals and communities. Some data suggests that greenspace exposure may moderate income-related health inequalities (49, 50), but inequality of access to nature could exacerbate these differences (51–53). Addressing these barriers to access would, therefore, be an essential aspect of developing successful NBHT interventions, and doing so will involve taking into account highly localised factors ranging from transport inequality (54) to the politics of land ownership and access rights (55).

For example, in a European context, there exists a wide spectrum of legal frameworks for access to natural areas (56). Although improving access is an important goal in regions with poorer access rights, in colonised regions such as Australia and Canada, improving access to land under the stewardship of Aboriginal peoples must be done under the leadership of and with deep sensitivity to First Nations practices and knowledge (57, 58) or run the risk of perpetuating a form of eco-colonialism (59). Nicholls (60, 61) has cautioned that the movement towards the BPS model and enactive models in MSK care could perpetuate the entrenched tendency in neoliberal politics towards promoting individual responsibility over state or collective action. Developing or promoting NBHT interventions without these political or social considerations for disadvantaged people may reflect this, while lobbying for public transport options and access rights could address these inequalities.

On an individual level, the relationship between people and nature is subject to influences ranging from cultural values and norms (62) to psychological traits such as nature connectedness (63), and these are likely to influence outcomes arising from interactions with natural spaces. One of the purported goals of NBHT is to increase nature connectedness due to an associated increase in pro-environmental behaviour (63). Nature connectedness may be seen to increase with both repeated nature engagements (64) or a singular meaningful experience (65). While high levels of nature connectedness may result in a more positive response to natural environments, nature connectedness may also be associated with eco-anxiety, which can indicate poorer mental health (66, 67). Although there is nuance about how eco-anxiety may be constructive or unconstructive with regard to pro-environmental behaviours (68, 69), it is important to acknowledge that the psychological effects of NBHT are likely to be highly variable even amongst those with a pro-nature disposition (70, 71).

## New directions and considerations

### Meaning in nature connection

While there is a low proportion of high quality primary studies in the field, NBHT programmes are being developed, promoted, and integrated into health management by governments, local communities, and healthcare providers (9). More granular data may suggest how NBHT could improve health outcomes in specific populations and contexts, but the inherent uncertainty of the BPS/enactive models and the highly personal relationship which people have with the natural world make it difficult to predict or quantify outcomes for individuals. However, patient values are considered to be of significant importance in contemporary MSK research (30, 72), and NBHT may offer an opportunity for patients to engage in valued nature-based activities. Although MSK professionals may be more comfortable exploring nature-related values with patients from the perspectives of potential biomedical benefits, changing contextual factors in the BPS model, or enactive-ecological opportunities for behavioural change, it is important to be able to explore the roles of personal meaning and connection with nature. A relationship with nature can bring people closer to their ‘inner world’ (73) and may be a means of attuning to the processes of life in various philosophical, spiritual, or religious traditions (74, 75). NBHT programmes that approach this subject from a pluralistic perspective will allow more space for individuals and groups to explore their relationship with nature in a manner connected to their respective frames of understanding.

### Ecological disturbance

One issue which has not been addressed in the NBHT literature is the potential risk of ecological disturbance. Engaging with natural spaces is framed as a positive action, but research in the field of ecotourism is showing that human interactions motivated by a desire to connect with biodiverse spaces can have adverse effects (76–78). Particular species and ecosystems may be more vulnerable to ecological disturbance than others (79–81), and it is important to consider whether NBHT interventions could exacerbate or mitigate this. For NBHT programmes to benefit both people and wildlife, management strategies informed by local ecological knowledge and adapted from the related field of ecotourism (78) can help to minimise disturbance to non-human species via strategies ranging from education initiative to community outreach programmes (82, 83).

### Anthropocentrism

Although NBHT may have significant potential for human health and carry-potential co-benefits for the environment through a variety of pathways, it should also be noted that it carries the risk of perpetuating anthropocentrism due to its principal focus on human health. Primary concern for human interests and benefits has been extensively criticised as one of the root causes of environmental degradation since colonial ages. To counteract this tendency in recent developments across planetary health, One health, and even conservation, the argument is made that a stronger concern for the environment, acknowledging the intrinsic value of non-human existence and seeing humans not as dominant but part of nature, needs to ground all initiatives, from local actions to global policy (60, 84–88). To this end, the development of NBHT should, at a minimum, always be reflective of its understanding of nature and the human-nature relationship, and strive to produce ecological surplus value beyond narrowly defined human health interests.

## Critical considerations for NBHT programming and prescription

To help aid the development of NBHT programmes by healthcare professionals, the following is a provisional set of critical considerations that may help in designing, implementing, and prescribing interventions that align with the evidence and help to address some of the challenges outlined in this perspective. These values are not exhaustive, nor are they likely to be universally applicable. It is the intention of the authors that these values are continuously updated to reflect the growing evidence and theoretical base of NBHT, and are adjusted and tailored to focus on the reality of a given social, environmental, cultural and political context.

1. Locality

NBHT should ideally be based on what is most local, minimising transport emissions, while maximising the opportunities for people to develop relationships with their environment.

1. Social and economic

The social and economic challenges faced by an individual/community should be taken into account when developing/prescribing NBHT programmes.

1. Benefits and outcomes

Claims in relation to benefits and outcomes must be aligned with the evidence base – it is especially important that studies on asymptomatic populations are not extrapolated to those with clinical conditions.

1. Shared multidisciplinary ecological knowledge

In order to minimise ecological disturbance, NBHT programmes should seek the knowledge of environmental/conservation groups to establish a balance between access to sites and potential disturbance of sensitive ecological processes.

1. Access and activism

Healthcare professionals may need to leverage the power of their communities, professional bodies, etc., to lobby for sustainable transport options for potential NBHT programmes and/or democratic engagement when land-use and ownership is a barrier to access of suitable natural sites.

1. ‘Leave no trace’

The roll-out of NBHT programmes must be accompanied by the promotion of codes of ethical and behavioural conduct to respect non-human life in a given setting.

1. Respecting indigenous peoples

In areas of historical or ongoing colonialism, respect for local knowledge and the leadership of local communities should be an absolute prerequisite for NBHT programme development, grounded in efforts to return respective regions to their pre-colonial owners and, therewith, indigenous stewardship.

1. Pluralism

The beliefs, psychological traits, and cultural values through which people relate to nature are highly varied, and NBHT programmes should seek to be cognisant of this.

1. Training and education

An introduction to NBHT application and prescribing can be developed in undergraduate and postgraduate MSK education.

## Conclusion

In this paper, we have outlined some of the key challenges in the research, application, and theoretical underpinnings of NBHT. The quality of many studies is poor and has often been conducted through a reductionist biomedical frame. NBHT may have significant effects on conditions better understood through the BPS model or enactive-ecological models of health and disease, but as a complex intervention, it requires a nuanced understanding of how it may apply to a given individual or group. Health professionals such as physiotherapists, osteopaths, occupational therapists, etc., may be ideally suited to develop NBHT programmes in their localities and communities as part of an effort to improve and connect public and planetary health. Doing so requires sharing multidisciplinary knowledge that addresses barriers to nature access and engagement and prioritising the concomitant protection/expansion of ecological processes and the non-human world. We conclude by offering a set of critical considerations aimed at helping healthcare professionals navigate the complexity of developing NBHT programmes that consider the best interests of people and nature in a range of social and environmental contexts.

## Data Availability

The original contributions presented in the study are included in the article/supplementary material, further inquiries can be directed to the corresponding author.
